# Multiomic analysis of CCNE1 amplification associated molecular and immune features in gynecological cancers

**DOI:** 10.1007/s12672-025-04032-7

**Published:** 2025-12-03

**Authors:** Linlin Liu, Wenjin Zheng, Liang Wen, Zhe Zhang, Yuanguang Meng

**Affiliations:** 1 Senior Department of Gynecology and Obstetrics, Seventh Medical Center of Chinese PLA General Hospital, Beijing, 100700 China; 2 Department of Gynecology and Obstetrics, The First Medical Center of PLA General Hospital, Beijing, People’s Republic of China

**Keywords:** CCNE1, Ovary, Uterus, Cervix, Carcinoma, Genomics

## Abstract

**Purpose:**

To delineate the genomic and cellular features of CCNE1 amplification across gynecologic cancer types using large-scale multi-omics data and assess its relevance for patient stratification and therapeutic targeting.

**Methods:**

We analyzed genomic and transcriptomic data from 12,845 patients with gynecological cancers curated from the cBioPortal database, including ovarian, endometrial, and cervical carcinomas. CCNE1 amplification rates, cooccurring genomic alterations, and pathway enrichment were evaluated. Single-cell RNA sequencing (scRNA-seq) and spatial transcriptomic data from cervical, endometrial, and ovarian tumors were used to investigate CCNE1 expression patterns, cell type specificity, and microenvironmental interactions.

**Results:**

CCNE1 amplification was detected in 8.40% of all patients, with the highest rates in patients with ovarian cancer (8.01%) and endometrial cancer (5.06%). Among the ovarian subtypes, carcinosarcoma showed the highest amplification frequency (12.46%), followed by high-grade serous carcinoma (9.84%). Amplified tumors are frequently co-altered by TP53, PIK3CA, and PI3K-AKT pathway genes. Single-cell analyses revealed that CCNE1 expression was largely confined to epithelial cells and was associated with proliferative and structural remodeling pathways, with limited immune engagement across tumor types. Spatial transcriptomics confirmed the localization of CCNE1 in epithelial-rich regions and its correlation with adhesion- and motility-related genes in clear cell and high-grade serous ovarian carcinomas.

**Conclusion:**

CCNE1 amplification is enriched in aggressive histologic subtypes of gynecological cancers and is linked to epithelial-specific expression and immune exclusion. These findings indicate that CCNE1 represents a potential molecular marker for tumor aggressiveness, warranting further investigation in the context of patient stratification and therapeutic targeting.

**Supplementary Information:**

The online version contains supplementary material available at 10.1007/s12672-025-04032-7.

## Introduction

Gynecological cancers, especially cervical carcinoma (CC), endometrial carcinoma (EC) and ovarian carcinoma (OC), seriously threatens female mortality, with increasing mortality rates [1]. Genomic copy number alterations (CNAs) are the major oncogenic drivers of these malignancies [2–4]. Amplification of the cyclin E1 gene (CCNE1, 19q12) is the key genetic event driving CCNE1 overexpression, occurring in approximately 20% of high grade serous ovarian carcinomas (HGSOC) [5, 6], 10–15% of uterine serous and carcinosarcoma cases [7, 8], and 15% of clear cell carcinomas (CCOC) [9]. Overexpression of CCNE1 elevates replication stress and genomic instability by accelerating the G1/S phase transition, which in turn confers primary resistance to platinum and PARP inhibitors [10–12].

Pharmacological vulnerabilities in CCNE1-amplified tumors have been increasingly recognized. A phase II trial of the inhibitor WEE1 kinase (adavosertib) demonstrated durable responses in CCNE1-amplified, platinum-resistant HGSOC: a 36% objective response in ovarian cases (ORR) and a median PFS of 6.3 months [13]. Additionally, mTOR pathway inhibitors, such as everolimus and vistusertib, have been reported to reduce tumor growth in CCNE1-amplified ovarian xenografts and increase their sensitivity to PARP inhibitors [14]. Multiple novel agents targeting CCNE1-amplified tumors, either as monotherapies or in combination with platinum agents, are currently under investigation, underscoring the importance of profiling CCNE1 status for personalized treatment decisions across gynecological malignancies [15–17].

Here, integrating multi-omic data enabled a deeper understanding of the cellular architecture and functional dependencies driving CCNE1-amplified tumors. In this study, we combined bulk CNA analysis, single-cell transcriptomics, and spatial transcriptomics across > 12,000 gynecological tumor samples to characterize the heterogeneity of CCNE1 amplification. Our analysis revealed how CCNE1-high epithelial subsets contribute to tumor progression and immune evasion, providing a rationale for the development of histotype-specific therapeutic strategies for selecting patient subsets.

## Methods

Bulk genomic data were obtained from the American Association of Cancer Research (AACR) Project Genomics Evidence Neoplasia Information Exchange (GENIE) (version 13.1) and accessed via cBioPortal (https://www.cbioportal.org). The publicly available dataset contained anonymized genomic profiles from patients with diverse cancer types collected from 19 contributing institutions. All participating institutions used next-generation sequencing assays to detect somatic mutations in formalin- or paraffin-fixed tumor specimens. Target enrichment was achieved through either hybrid capture or amplicon-based PCR by sequencing panels of varying sizes. Copy number alterations were observed in a subset of the cases. Patients diagnosed with ovarian (including high-grade serous, low-grade serous, endometrioid, mucinous, carcinosarcoma, and clear cell subtypes), uterine (endometrioid, serous, carcinosarcoma, and clear cell), and cervical (squamous, adenocarcinoma, and adenosquamous) carcinomas were selected using the cBioPortal platform. Both primary and metastatic tumor samples were included; however, for patients with multiple sequenced specimens, only one representative sample (preferably from the primary site) was retained to avoid duplication. Cases lacking complete copy number alteration (CNA) profiles or key clinical annotations were excluded. CCNE1 amplification rates were then calculated overall and stratified by tumor site and histological subtype. Co-occurring genomic alterations in CCNE1-amplified tumors were also investigated. Variant functional annotation and classification of pathogenic alterations were performed using OncoKB [18], which is integrated into the cBioPortal platform. Because the AACR GENIE cohort integrates data from multiple sequencing panels across contributing institutions, minor inter-panel variability in copy number detection may exist. CCNE1 amplification calls were extracted as reported in the harmonized GENIE dataset, which applies standardization and QC pipelines to minimize batch effects across panels. High-level amplification was defined based on GISTIC copy number calls, with values ≥ + 2 considered as amplified. No additional batch correction was applied beyond the harmonization performed by GENIE.

To investigate the role of CCNE1 amplification across different gynecologic cancer subtypes, single-cell RNA sequencing (scRNA-seq) datasets related to cervical, endometrial, and ovarian cancers were retrieved from the GEO database (https://www.ncbi.nlm.nih.gov/geo/). From the ovarian cancer dataset GSM235931 (*n* = 15), only samples derived from primary ovarian tumor tissues were selected, including three high-grade serous ovarian carcinoma (HGSOC) samples, two low-grade serous ovarian carcinoma (LGSOC) samples, two carcinosarcoma samples, two clear cell carcinoma (CCOC) samples, and two endometrioid carcinoma samples. The cervical cancer dataset GSE197461 consisted of scRNA-seq data from three squamous cell carcinoma (GSM5917937–GSM5917939) and three adenocarcinoma (GSM5917940–GSM5917942) cases. Owing to the limited availability of single-cell data for endometrial cancer subtypes, three endometrioid endometrial carcinoma samples (GSM5276933–GSM5276935) from the GSE173682 dataset were included in the analysis. Cervical cancer samples were generated using the Illumina NovaSeq 6000 platform (GPL24676), whereas the ovarian and endometrial cancer samples were sequenced using the Illumina NextSeq 500 platform (GPL18573). All datasets were provided in 10X Genomics-compatible format, allowing uniform preprocessing and integration via the Seurat package for downstream single-cell analysis.

Additionally, single-cell RNA-seq data were processed via the Seurat package, and batch effects across samples were addressed using the harmony algorithm. Cell type annotation was subsequently performed using the SingleR package with the Human Primary Cell Atlas (HPCA) dataset as the reference for label transfer. The mitochondrial gene content was computed via the percentage feature set function, and the low-quality or damaged cells with extreme gene counts (< 50 or > 9,000) and those with mitochondrial transcript fractions exceeding 15% were filtered out. Standard preprocessing steps—including normalization, scaling, and principal component analysis (PCA)—were performed. The number of principal components used for downstream analysis was set to 15 based on ElbowPlot. Dimensionality reduction via t-distributed stochastic neighbor embedding (t-SNE) was applied to visualize the cellular distributions. Following cell type annotation using the SingleR package, major cell populations, including T cells, epithelial cells, neutrophils, macrophages, B cells, fibroblasts, NK cells, and endothelial cells, were identified. Intercellular communication analysis was subsequently performed using CellChat, with a focus on signaling interactions between epithelial cells and other cell types. Normalized but uncorrected expression data were used as input for CellChat to preserve biologically relevant ligand–receptor signals. Epithelial cells were identified by canonical markers (EPCAM/KRTs) after SCTransform normalization and Harmony integration. Within each sample, CCNE1^high^ epithelial cells were defined as the top quartile (≥ 75th percentile) of scaled CCNE1 expression, and CCNE1low as the bottom quartile (≤ 25th percentile); cells with intermediate expression were excluded from binary comparisons. Differential gene expression between the CCNE1^high^ and CCNE1^low^ groups was assessed via the FindMarkers function in Seurat, with thresholds set at |avg_log2FC| >2 and adjusted p-value < 0.05. Pathway enrichment of the DEGs was performed using Gene Ontology (GO) and Kyoto Encyclopedia of Genes and Genomes (KEGG) analyses. Enriched terms with adjusted p values < 0.05 were considered significant, and the top 10 enriched terms from each GO category and KEGG pathway analysis were selected for display. The tumor mutational burden and immune cell composition between groups were evaluated using the maftools and CIBERSORT algorithms in R and visualized using the ggplot2 package.

Spatial transcriptomic data from CCOC (GSM7019835) and HGSOC (GSM8207499) samples were retrieved from the GEO database. These datasets were selected because they represent the only publicly available high-quality spatial transcriptomic profiles of ovarian cancer subtypes with corresponding histopathological annotations at the time of analysis. Raw gene-spot matrices were processed using the Seurat package, and normalization across spatial barcodes was carried out using the SCTransform function to ensure comparability across tissue sections. Spatial-level expression and visualization of CCNE1 and other cell type-specific markers were performed using the SpatialFeaturePlot and FeaturePlot functions in R. Spearman correlation and Euclidean distance-based analysis were used to evaluate spatial colocalization between CCNE1 and immune or structural markers. To evaluate the statistical significance of spatial proximity, we generated randomized controls by permuting spatial coordinates 1,000 times within each tissue section while maintaining the overall expression distribution. High-expression regions were defined as the top 25% of CCNE1 expression, and proximity significance was assessed against randomized controls. A genome-wide screen further identified genes with consistently close spatial proximity to CCNE1^high^ regions.

All the statistical analyses were performed in R (v4.0.5) using the Seurat, ggplot2, dplyr, and clusterProfiler packages. Group comparisons of gene expression, immune cell composition, and tumor mutational burden were conducted using the Wilcoxon rank-sum test or the chi-square test, as appropriate. To control for multiple hypothesis testing, P values were adjusted using the Benjamini–Hochberg false discovery rate (FDR) procedure unless otherwise specified. Pathway enrichment was performed with clusterProfiler (enrichGO/enrichKEGG) using BH correction (p.adjust < 0.05), and results are reported as adjusted P values (q values). Spatial correlation and proximity between CCNE1 and selected marker genes were evaluated using Spearman correlation and Euclidean distance metrics, with significance assessed using the Mann‒Whitney U test against randomized controls. Statistical significance was set at *p* < 0.05.

## Results

### CNA profiling across gynecological cancers

**Fig. 1:**
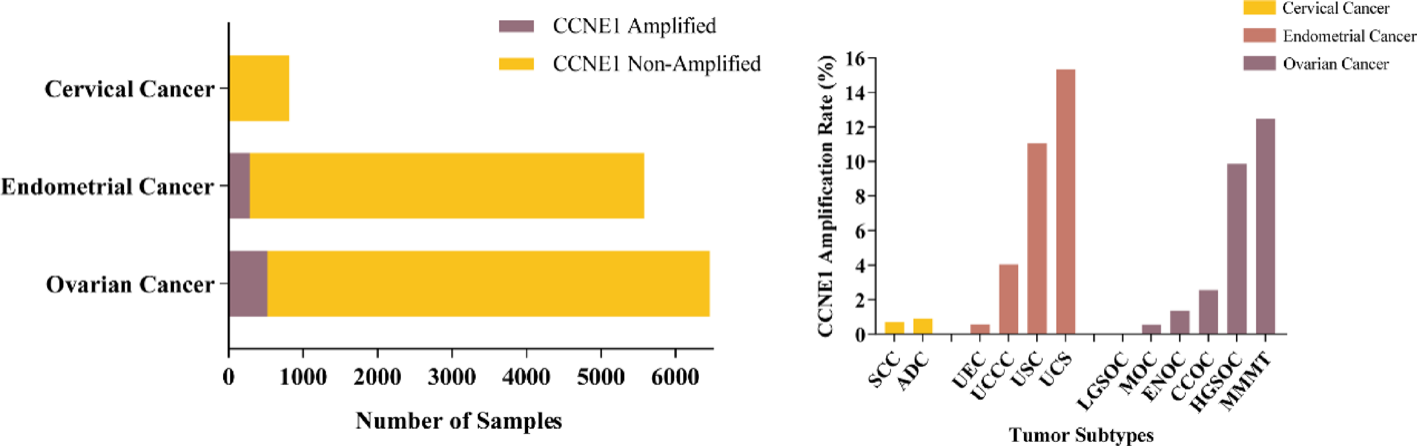
CCNE1 amplification landscape in gynecological cancers. **A** Distribution of CCNE1 amplification among cervical, endometrial, and ovarian cancers on the basis of data from 12,845 patients in the cBioPortal database. **B** CCNE1 amplification rates across histological subtypes, with the highest frequencies observed in ovarian carcinosarcoma, high-grade serous carcinoma, and uterine serous carcinoma. *SCC* squamous cell carcinoma, *ADC* adenocarcinoma, *UEC* uterine endometrioid carcinoma, *UCCC* uterine clear cell carcinoma, *USC* uterine serous carcinoma, *UCS* uterine carcinosarcoma, *LGSOC* low-grade serous ovarian carcinoma, *MOC* mucinous ovarian carcinoma, *ENOC* endometrioid ovarian carcinoma, *CCOC* clear cell ovarian carcinoma, *HGSOC* high-grade serous ovarian carcinoma, *MMMT* ovarian carcinosarcoma

A cohort of 12,845 eligible patients was identified by searching the Cbioportal database, with ovarian, endometrial, and cervical cancer cases comprising 50.25%, 43.39%, and 6.35% of the group, respectively. The median age at the time of sample acquisition was 63 years (range: 18–89 years). The majority of the patients were white (75.34%), with non-Hispanic white individuals comprising the largest racial group (73.11%). A total of 62.5% of the samples were obtained from primary tumors. Overall, CCNE1 amplification was identified in 1,079 of the 12,845 patients (8.40%). When patients were stratified by tumor site (Fig. 1A), the amplification rate was approximately 0.74% for cervical carcinomas, 5.06% for endometrial carcinomas, and 8.01% for ovarian carcinomas (*P* < 0.05). Other common genomic CNVs identified in patients with gynecological malignancies included amplification of MYC (5.7%), TERC (5.4%), AGO2 (4.8%), and ERBB2 (3.7%), as well as homozygous deletions in EIF1AX (2.2%), CDKN2A (2.1%), and CDKN2B (1.9%). The mean frequency of CCNE1 gene amplification in metastatic tumors was 8.12%, whereas that in primary tumors was 5.86% (*P* < 0.05). Among the samples with CCNE1 amplification, co-amplified genes, including AKT2 (22.24%), MYC (10.19%), ERBB2 (8.79%), and KRAS (7.58%), were predominantly enriched in the PI3K-AKT pathway. Moreover, pathogenic gene mutations predominantly involved TP53 (71.46%), PIK3CA (14.29%), and PIK3R1 (5.84%), which are associated with P53 and PI3K-AKT pathways.

**Fig. 2:**
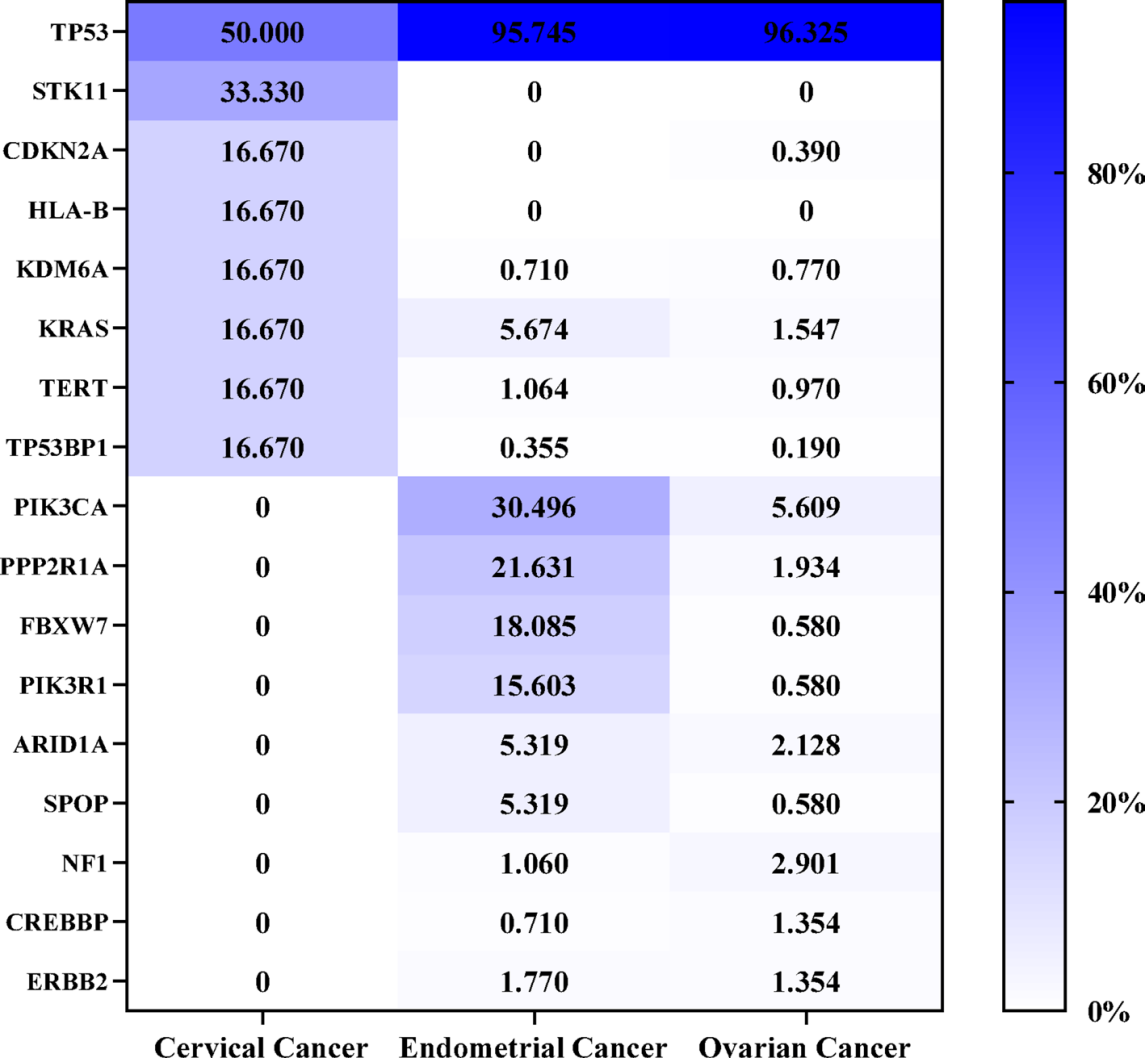
Frequency of cooccurring pathogenic mutations in CCNE1-amplified gynecologic tumors. Heatmap showing the mutation frequency of key cancer-related genes in CCNE1-amplified gynecological tumors. Mutation frequencies were color-coded from low (white) to high (dark blue)

**Fig. 3:**
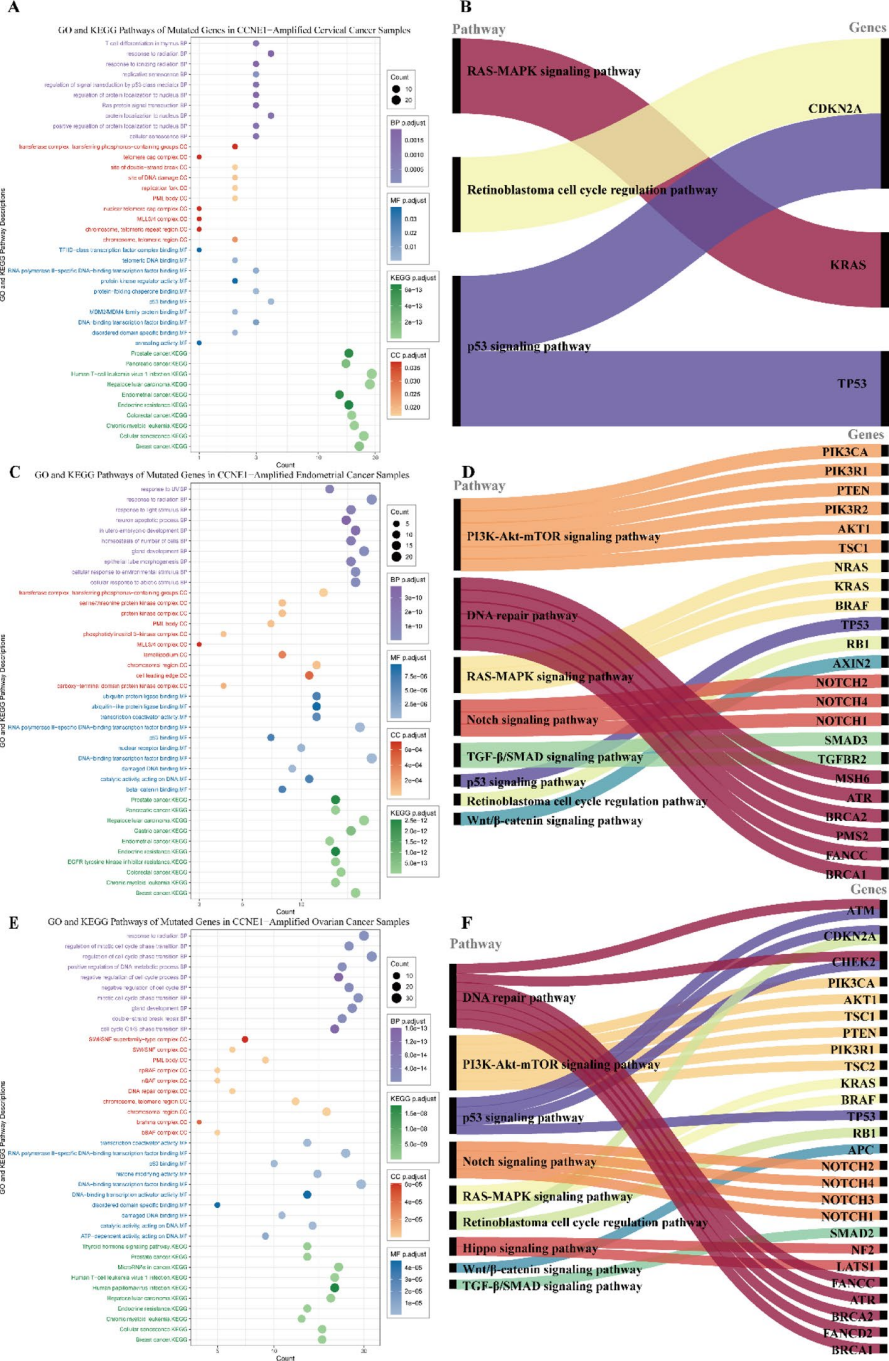
Pathway enrichment analysis of mutated genes in CCNE1-amplified gynecological cancers. **A**–**B** In cervical cancer, mutated genes were enriched in the p53, RAS-MAPK, and retinoblastoma pathways, with co-mutated genes such as TP53 and KRAS mapped to key oncogenic signaling. **C**–**D** In endometrial cancer, enrichment was observed in the PI3K-Akt-mTOR, DNA repair, and Notch pathways, as shown by the distribution of relevant co-mutated genes. **E**–**F** In ovarian cancer, mutated genes are involved in cell cycle regulation and multiple hallmark pathways, including Hippo and p53 signaling, with key genes linked to DNA repair and RAS-MAPK signaling

In cervical cancer patients, CCNE1 amplification occurred in 0.69% of squamous cell carcinomas (SCC, 3/434) and 0.91% of adenocarcinomas (ADC, 3/331), with no amplification detected in adenosquamous carcinomas (0/51; Fig. 1B). Among patients with CCNE1 amplification, 50% also harbored a TP53 mutation, followed by STK11 mutations in 33% of cases (Fig. 2). Pathway enrichment analysis of these mutated genes revealed several oncogenic signaling pathways, including p53 regulation, Ras protein signal transduction, and cellular senescence, as well as tumor immunity pathways related to T-cell differentiation and the regulation of protein localization to the nucleus, with most of which were enriched in hallmark cancer pathways, including p53, RAS-MAPK, and retinoblastoma signaling (Fig. 3A and B).

In patients with endometrial cancer, the overall incidence of CCNE1 amplification was 5.06% (282/5574, Fig. 1A), with significant variation across histological subtypes (*P* < 0.05, Fig. 1B), including the highest rate in uterine carcinosarcoma (15.35%, 122/795), followed by uterine serous carcinoma (11.05%, 133/1204), clear cell carcinoma (4.04%, 8/198), and endometrioid tumors (0.56%, 19/3377). The molecular profile of CCNE1-amplified endometrial tumors revealed a predominance of pathogenic genomic alterations, with the most frequently affected genes being TP53 (95.74%), PIK3CA (30.50%), PPP2R1A (21.63%), FBXW7 (18.09%), PIK3R1 (15.60%), and KRAS (5.67%), as shown in Fig. 2. The results of the pathway enrichment analysis revealed that these mutated genes are involved in p53 binding, nuclear receptor binding, and phosphatidylinositol 3-kinase complex activity and are enriched mainly in cancer-related pathways, such as the PI3K-Akt-mTOR, DNA repair, p53, and Notch signaling pathways, along with processes related to cell morphology and the environmental response (Fig. 3C and D).

CCNE1 amplification was identified in 8.01% of the patients with ovarian cancer (Fig. 1A). Among the various histological subtypes (Fig. 1B), the highest incidence was observed in carcinosarcoma at 12.46% (35/281). Amplification rates were also detected for high-grade serous carcinoma (9.84%, 463/4703), clear cell carcinoma (2.55%, 13/509), endometrioid carcinoma (1.36%, 5/369), and mucinous ovarian carcinoma (0.53%, 1/187). No CCNE1 amplification was detected in low-grade serous carcinoma. In patients with CCNE1-amplified tumors, the most frequently observed pathogenic genomic alterations were mutations in TP53 (96.32%), PIK3CA (5.61%), NF1 (2.90%), ARID1A (2.12%), and PPP2R1A (1.93%). GO and KEGG pathway analyses (Fig. 3E and F) revealed that these mutated genes were significantly enriched in cell cycle processes (G1/S transition, DNA repair; FDR < 0.05) and oncogenic pathways such as PI3K-Akt-mTOR, p53, Notch, and Hippo signaling.

### Single-cell analysis in cervical cancer

**Fig. 4:**
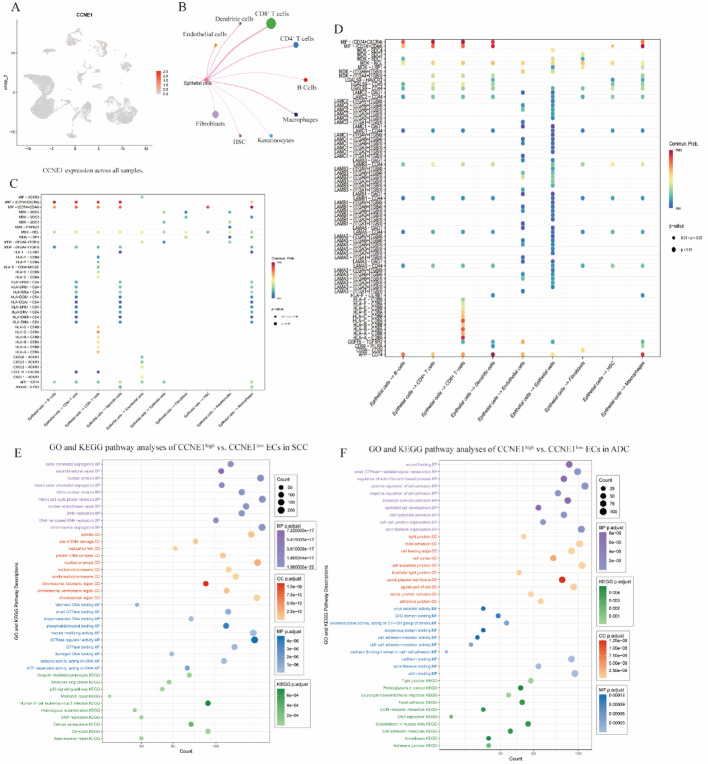
Single-cell transcriptomic analysis of CCNE1 expression and the microenvironmental context of cervical cancer subtypes. **A** UMAP visualization of CCNE1 expression levels across all single-cell profiles, highlighting enrichment in epithelial cells. **B** Cell‒cell communication network showing interactions between epithelial cells and immune cell types, especially CD8⁺ T cells, through HLA‒CD8 signaling. **C** Interaction heatmap in SCCs showing epithelial cell-derived proinflammatory and immune-evasive ligand‒receptor pairs. **D** Interaction heatmap of the ADC highlighting epithelial-driven structural and immunosuppressive signals. **E** GO and KEGG pathway enrichment analysis of CCNE1^high^ vs. CCNE1^low^ epithelial cells in SCC, showing enrichment in T-cell activation and viral response pathways. **F** GO and KEGG pathway analyses of CCNE1^high^ vs. CCNE1^low^ epithelial cells in ADC, indicating their involvement in adhesion, matrix remodeling, and proliferation. *SCC* squamous cell carcinoma, *ADC* adenocarcinoma, *CC* cervical carcinoma, *ECs* epithelial cells

To resolve cellular heterogeneity, we analyzed single-cell RNA-seq data from three SCCs and three ADC tumors (GSE197461) to map CCNE1 expression and its microenvironmental interactions. Following quality control (nFeature_RNA > 50 and mitochondrial fraction < 15), principal component analysis (PCA) confirmed the absence of batch effects across samples (Supplementary Figure S1A). Subsequent t-SNE clustering identified 27 distinct cellular subclusters, which were further annotated using the SingleR package, resulting in nine and eight major cell types in SCC and ADC cases (Supplementary Figure S1B), respectively. Ten major cell types were characterized across all six cervical cancer cases: CD8^+^ T cells, epithelial cells, macrophages, B cells, fibroblasts, hematopoietic stem cells (HSCs), keratinocytes, and dendritic and endothelial cells (Fig. 4A). Although CCNE1 was not broadly upregulated across the samples, high-expression clusters were predominantly confined to epithelial cells (ECs) in both subtypes (Fig. 4B). Cell‒cell communication analyses revealed prominent EC‒CD8⁺ T cell interactions mediated by multiple HLA class I-CD8 receptor pairs (HLA‒A/B/C‒CD8A/CD8B) (Fig. 4B). Nevertheless, SCC ECs (Fig. 4C) demonstrated greater enrichment of proinflammatory and immune-evasive ligands (CXCL1-ACKR1, CXCL8-ACKR1, and MIF-CD74/CXCR4), whereas ADC ECs (Fig. 4D) engaged more extensively in structural and immunosuppressive signaling (laminin-integrin interactions, LGALS9-HAVCR2, and GDF15-TGFBR2). Consistent with these findings, GO and KEGG pathway analyses of CCNE1^high^ vs. CCNE1^low^ ECs revealed shared enrichment of proliferative pathways (cell cycle, DNA replication, and p53 signaling) and subtype-specific differences, including T-cell receptor/viral infection pathways in SCC versus cell adhesion and extracellular matrix remodeling in ADC (Fig. 4E and F).

### Single-cell analysis in endometrial cancer

**Fig. 5:**
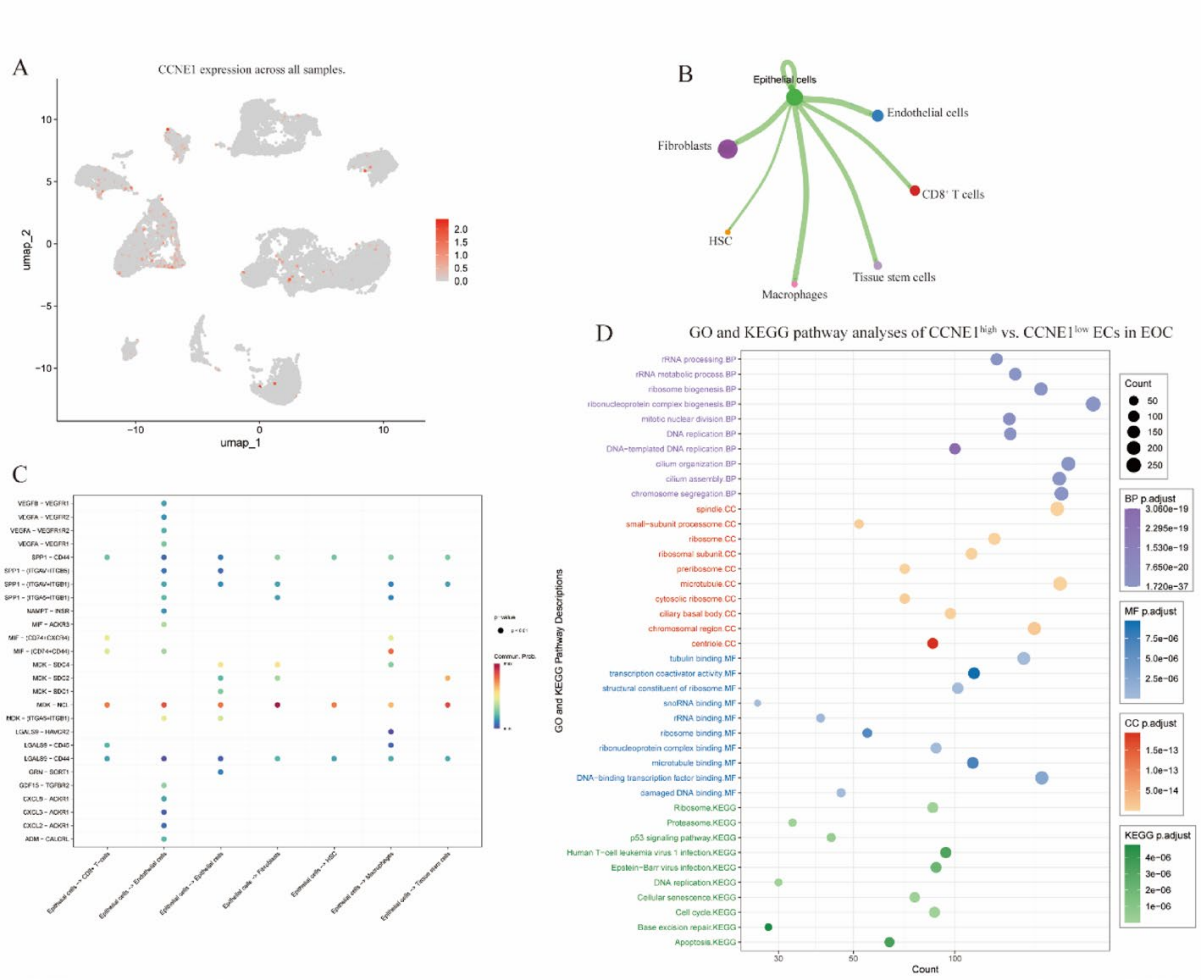
Single-cell transcriptomic profiling of CCNE1 expression and cellular interactions in endometrial carcinoma. **A** UMAP visualization of CCNE1 expression across all cells, showing enrichment primarily in epithelial clusters. **B** Cell‒cell interaction network identifying epithelial cell communication with endothelial cells, fibroblasts, and other stromal components. **C** Heatmap of key ligand‒receptor pairs mediating epithelial interactions, particularly the VEGFA‒VEGFR and MDK‒NCL axes. **D** GO and KEGG pathway enrichment analysis of CCNE1^high^ vs. CCNE1^low^ epithelial cells. *EOC* endometrial cancer, *ECs* epithelial cells

Single-cell RNA-seq data (GSM173682) from three endometrial carcinoma samples were analyzed under standard quality control (nFeature_RNA > 50 and percentage. mt < 15, Supplementary Figure S1C), PCA, and t-SNE clustering procedures, yielding 20 cell clusters. These clusters were annotated into seven major cell types by SingleR (Supplementary Figure S1D): CD8 + T cells, epithelial cells, macrophages, fibroblasts, HSCs, tissue stem cells and endothelial cells. Although CCNE1 was not broadly upregulated across the samples, high-expression clusters were predominantly localized in epithelial cells (Fig. 5A). In cell‒cell communication analyses (Fig. 5B‒5 C), robust epithelial‒endothelial/fibroblast interactions were observed, which were primarily mediated through the VEGFA‒VEGFR and MDK‒NCL axes associated with cell proliferation, adhesion, and angiogenesis. In addition, GO and KEGG pathway analyses of CCNE1^high^ versus CCNE1^low^ epithelial cells revealed significant enrichment in the proliferative and DNA repair pathways (Fig. 5D). However, no significant changes in immune composition or tumor mutational burden (TMB) were observed between CCNE1^high^ and CCNE1^low^ epithelial cells (Supplementary Figure S2).

### Single-cell and Spatial transcriptomic analysis in ovarian cancer

**Fig. 6:**
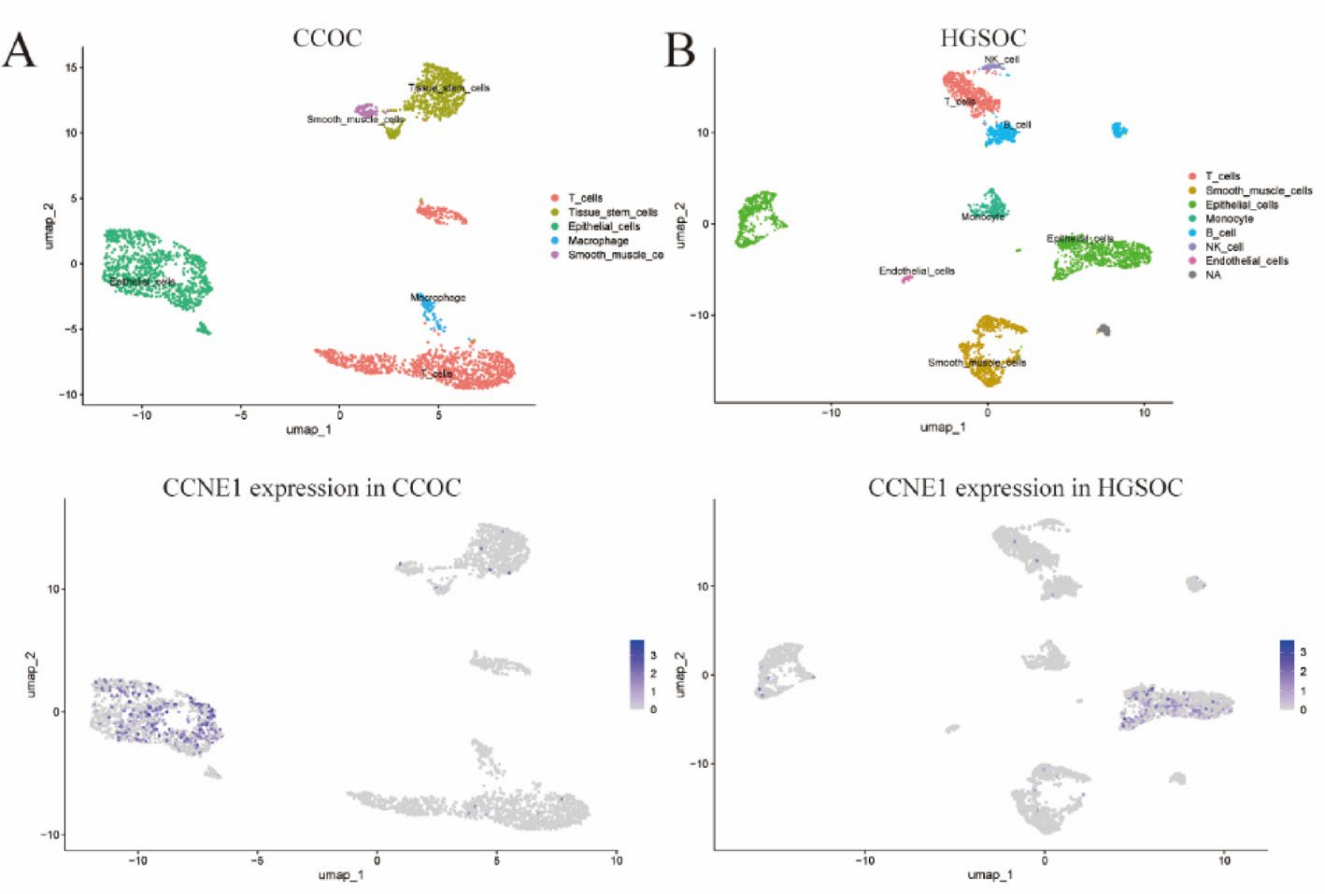
Single-cell RNA-seq analysis of CCNE1 expression in ovarian cancer subtypes. **A** UMAP plot and CCNE1 expression in clear cell ovarian carcinoma (CCOC), demonstrating epithelial cell-restricted expression. **B** UMAP plot and CCNE1 expression in high-grade serous ovarian carcinoma (HGSOC), similarly showing expression enrichment in epithelial clusters. *MMMT* ovarian carcinosarcoma, *COCC* clear cell ovarian carcinoma, *ENOC* endometrioid ovarian carcinoma, *HGSOC* high-grade serous ovarian carcinoma, *LGSOC* low-grade serous ovarian carcinoma

To further investigate the role of CCNE1 in ovarian cancer subtypes, single-cell RNA-sequencing data (GSM235931) were analyzed, including samples from HGSOC, low-grade serous ovarian carcinoma (LGSOC), CCOC, endometrioid ovarian carcinoma (ENOC), and ovarian carcinosarcoma (MMMT). After quality control (nFeature_RNA > 50, mt < 15; Supplementary Figure S1E), PCA, and t-SNE, 16 cell clusters were identified and annotated into 5–8 to major cell types per subtype (Supplementary Figure S3A-S3D). Given that CCNE1 expression was predominantly restricted to ECs in CCOC (Fig. 6A) and HGSOC (Fig. 6B), downstream analyses focused on these two subtypes.

**Fig. 7:**
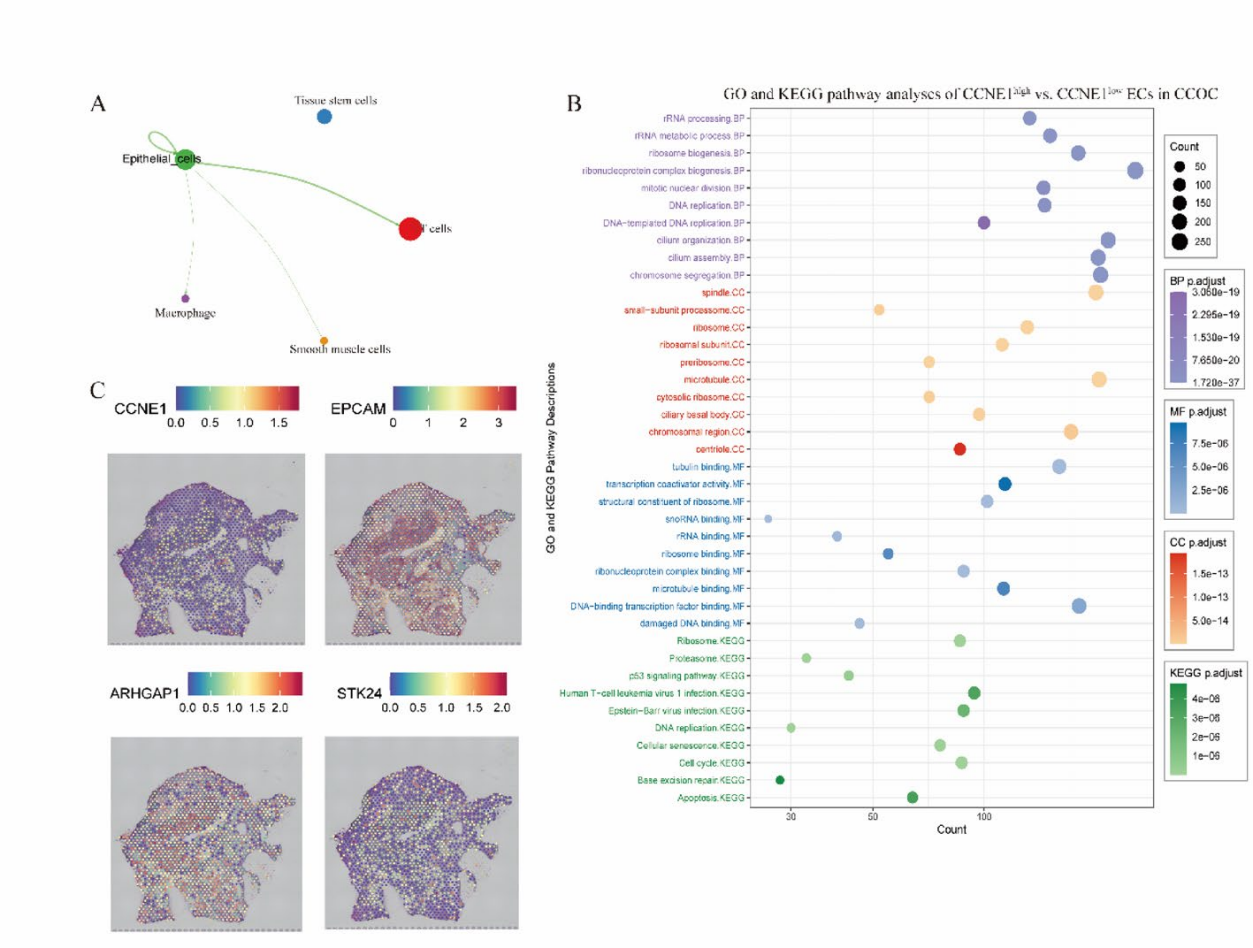
CCNE1-associated epithelial dynamics and spatial gene expression in CCOC. **A** Cell‒cell communication analysis showing limited epithelial cell (EC) interactions with other cell types in CCOC. **B** GO and KEGG pathway enrichment analyses of CCNE1^high^ versus CCNE1^low^ epithelial cells in CCOC revealed significant upregulation of pathways related to cell proliferation, adhesion, and cytoskeletal regulation. **C** Spatial transcriptomics (GSM7019835) depicting the gene expression patterns of CCNE1, EPCAM (epithelial marker), and the colocalized genes ARHGAP1 and STK24, which are involved in structural remodeling and cell motility. *ECs* epithelial cells, *CCOC* clear cell ovarian carcinoma

In the CCOC subset, ECs showed limited interactions with other cell types (Fig. 7A), and no significant differences in TMB or immune composition were observed between the CCNE1^high^ and CCNE1^low^ groups (Supplementary Figure S2). Nevertheless, CCNE1^high^ epithelial cells were enriched in the focal adhesion and wound healing pathways (Fig. 7B). To extend these findings to CCOC, spatial transcriptomic data (GSM7019835) were analyzed to visualize both global gene expression and CCNE1 distribution at the tissue level. CCNE1 was predominantly expressed in epithelial-rich regions (Fig. 7C), showing a moderate spatial correlation with the epithelial marker EPCAM (Spearman’s ρ = 0.52), in agreement with single-cell findings. Consistent with the scRNA-seq results, spatial proximity analysis revealed that CCNE1 was closely related to genes such as ARHGAP1 and STK24, which are involved in cytoskeletal remodeling and cell motility (Fig. 7C). In contrast, no notable spatial associations were detected between CCNE1 and immune-related genes such as PDCD1, CD274, CTLA4, CD8A, and GZMB (Spearman’s ρ between − 0.1 and 0.1) (Supplementary Figure S4). These findings suggest that CCNE1 may contribute to structural remodeling and local progression in CCOC, whereas its high-activity regions exhibit limited immune infiltration.

**Fig. 8:**
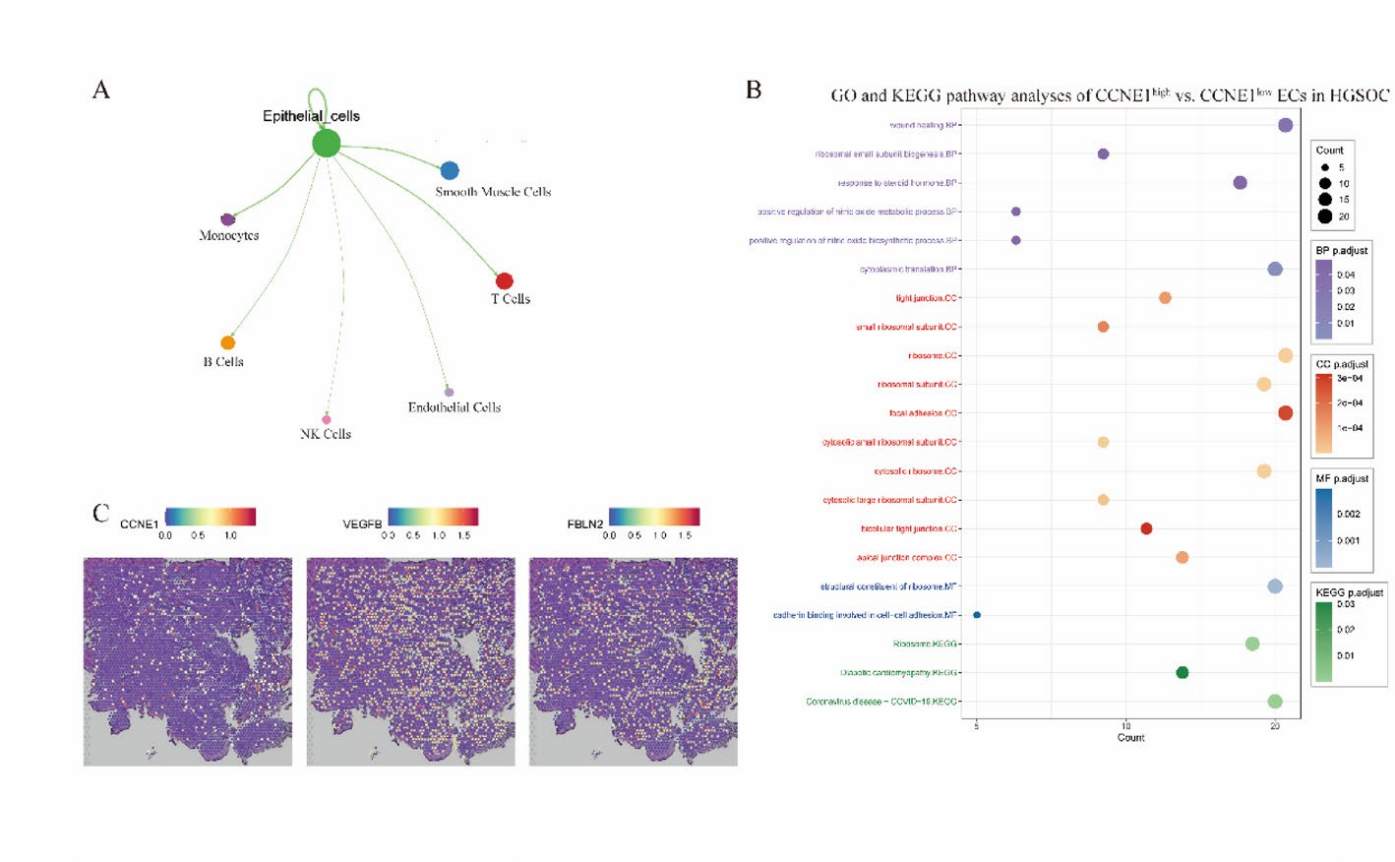
CCNE1-associated epithelial dynamics and spatial gene expression in HGSOC. **A** Cell‒cell communication analysis revealed limited interactions between epithelial cells (ECs) and other immune or stromal populations in HGSOC. **B** GO and KEGG pathway enrichment analyses comparing CCNE1^high^ and CCNE1^low^ ECs demonstrated the upregulation of cell adhesion and tight junction pathways. **C** Spatial transcriptomics (GSM8207499) revealed diffuse CCNE1 expression without distinct clustering, with elevated VEGFB and FBLN2 levels observed in CCNE1-high regions. *ECs* epithelial cells, *HGSOC* high-grade serous ovarian carcinoma

In the HGSOC subtype, limited cell‒cell communication was observed between ECs and other cell types (Fig. 8A). Further analysis of the differentially expressed gene pathways between the CCNE1^high^ and CCNE1^low^ groups revealed significant enrichment in tight junction and cell adhesion pathways (Fig. 8B), with no notable differences in TMB or immune composition between the groups (Supplementary Figure S2). To further explore these findings, spatial transcriptomic data from the GSM8207499 dataset were analyzed, and the distribution of CCNE1 expression was observed, as shown in Fig. 8C. However, these cells did not specifically cluster into a single group. Notably, in spatial regions with high CCNE1 expression, elevated expression of VEGFB and FBLN2, as well as enrichment in cell adhesion pathways, was observed (Fig. 8C). These results further support the role of CCNE1 in promoting structural remodeling and local progression in HGSOC.

## Discussion

We profiled CCNE1 amplification across 12,845 gynecologic tumors, revealing a site-specific prevalence—for example, 0.74% in cervical cancer, 5.06% in endometrial cancer, and 8.01% in ovarian cancers—and observed notable co-amplifications and mutations involving PI3K-AKT pathway genes and TP53. Single-cell RNA-seq and spatial transcriptomics confirmed that CCNE1 expression is largely restricted to epithelial cells, where CCNE1-high clusters exhibit proliferative signatures and structural remodeling while evading immune-cell interactions. These patterns are most pronounced in uterine carcinosarcoma (CCOC) and high-grade serous ovarian carcinoma (HGSOC). In contrast to previous TCGA and pan-cancer studies that focused on bulk-level genomic associations, this study integrates copy number, single-cell, and spatial transcriptomic data to define subtype-specific molecular and immune characteristics of CCNE1 amplification in gynecological cancers, providing higher spatial and cellular resolution than earlier analyses.

Our analysis revealed that CCNE1 amplification is rare in cervical cancer (< 1%), which is consistent with findings from a study [19] conducted in a low- and middle-income country, where CCNE1 expression was significantly upregulated during the progression from cervical intraepithelial neoplasia (CIN) to invasive carcinoma. These findings suggest that early cell cycle dysregulation may contribute to the malignant transformation of CC. Our single-cell RNA-seq data demonstrated that CCNE1 expression was localized predominantly localized in epithelial cell subsets of both squamous cell carcinoma (SCC) and adenocarcinoma (ADC). These findings suggest that CCNE1 activation is confined to discrete malignant epithelial subpopulations, consistent with the high transcriptional heterogeneity previously observed in epithelial cancer cells [20]. In cervical cancer, CCNE1 amplification may contribute to early epithelial transformation, potentially enhancing proliferative signaling in HPV-infected cells. This suggests that CCNE1 testing could complement existing HPV-based screening by identifying patients at higher risk of malignant progression.

Furthermore, cell‒cell communication analysis revealed distinct immune microenvironments between SCCs and ADCs. In SCC, epithelial cells interact frequently with CD8⁺ T cells through the HLA class I‒CD8 axes and secrete immunosuppressive ligands such as CXCL1‒ACKR1 and MIF‒CD74‒CXCR4, suggesting an immune-inflamed yet immunosuppressed phenotype. This finding aligns with a previous report [21] of SCC exhibiting immune infiltration along with localized immune evasion. In contrast, ADC epithelial cells primarily engaged in stromal and immunosuppressive signaling, including laminin–integrin and LGALS9–HAVCR2 interactions, which is consistent with an immune-excluded phenotype. This pattern is supported by recent findings [22] showing preferential expression of immune checkpoints such as HAVCR2 (TIM-3) in ADC-dominant immune-excluded microenvironments with reduced lymphocyte infiltration. Future studies using spatial multi-omics and functional assays are needed to validate these subtype-specific immune interactions and to explore their implications for targeted immunotherapy.

Our cohort revealed CCNE1 amplification in approximately 5–15% of high-grade and non-endometrioid ECs, which is comparable to the 22% frequency reported for the copy number-high subgroup in TCGA [23]. Similarly, in a large institutional cohort [24] of 2,042 ECs analyzed by targeted massive parallel sequencing, CCNE1 amplification was detected in 22% of cases, most of which were serous and carcinosarcoma. However, our observed frequency is lower than the 26.1% reported by Kuhn et al. via SNP array analysis [25] in uterine serous carcinoma (USC) and notably lower than the FISH-based estimate [26] of 45% and another ISH-based report [25] of 40.5%. These discrepancies likely reflect the methodological heterogeneity. Array-based comparative genomic hybridization (array-CGH) is a sensitive technique limited to predefined loci, whereas next-generation sequencing (NGS) offers broader coverage and higher resolution for novel CNV and breakpoint detection [27]. However, NGS findings require additional validation via orthogonal methods. Because our study utilized targeted NGS panels rather than genome-wide sequencing or FISH-based approaches, focal high-level amplifications of CCNE1 may be partially underestimated due to probe coverage limitations. This technical constraint should be considered when comparing our amplification frequencies with those derived from array-CGH or FISH assays. Our CCNE1 amplifications were derived from the AACR Project GENIE (v13.1), which utilizes targeted next-generation sequencing (NGS) panels, an approach that may underestimate focal amplification. Thus, the variations in CCNE1 amplification across studies may stem from technical factors, cohort composition, and inconsistent definitions of amplification.

Our single-cell analysis revealed that although CCNE1 expression was limited, it was concentrated in epithelial cells, reinforcing its role in cell cycle regulation and tumor proliferation. This pattern is consistent with prior findings [28] linking CCNE1 overexpression to genomic instability and aggressive behavior in endometrial cancer. Notably, epithelial–stromal communication through the VEGFA–VEGFR and MDK–NCL axes was prominent in the CCNE1^high^ clusters, suggesting coordination between proliferation and angiogenesis. Recent studies [29] have confirmed that VEGFA derived from epithelial and myeloid cells supports vascular remodeling in the endometrial tumor niche. These findings imply that anti-angiogenic agents such as bevacizumab or lenvatinib could be particularly effective in CCNE1-amplified subtypes exhibiting enhanced vascular remodeling.

Although CNE1^high^ epithelial cells were enriched in the cell cycle and DNA repair pathways, we did not observe major differences in immune composition or TMB, implying that the role of CCNE1 in early endometrial carcinogenesis may be driven more by stromal interactions and proliferative programming than by immune evasion.

Our analysis revealed that CCNE1 amplification occurred in 8.01% of ovarian cancer patients, with the highest frequency observed in carcinosarcoma patients (12.46%), followed by high-grade serous carcinoma patients (HGSOC patients, 9.84%). This distribution pattern is largely consistent with the results of previous genomic studies. For example, the Cancer Genome Atlas (TCGA) project identified CCNE1 amplification in approximately 20% of HGSOC patients, emphasizing its enrichment in this aggressive subtype [6]. While our frequency for HGSC is somewhat lower, this discrepancy may be due to differences in patient cohorts, bioinformatics thresholds, or sample sizes. Other studies have also supported the rarity of CCNE1 amplification in non-HGSC subtypes. Patch et al. [30] reported low frequencies of clear cells (3.3%) and endometrioid carcinomas (1.7%), which is consistent with our data on clear cells (2.55%) and endometrioid carcinomas (1.36%). Interestingly, our observation of no amplification in low-grade serous carcinoma (LGSOC) is consistent with the low genomic instability typically observed in LGSOC, which was also highlighted in a previous study [31].

In terms of co-occurring mutations, TP53 was altered in 96.32% of CCNE1-amplified tumors, which aligns with existing data showing that CCNE1 amplification is almost mutually exclusive with BRCA1/2 loss or HRD status but strongly co-occurs with TP53 mutations in HGSOC [32]. This co-alteration likely reflects a mechanistic synergy, as loss of p53-mediated G1/S checkpoint control permits unchecked CCNE1-driven cell cycle progression, thereby increasing replication stress and promoting genomic instability. Mutations in PIK3CA, ARID1A, and PPP2R1A were also present in smaller fractions, particularly in the non-HGSOC histology. Similar findings were reported by Wiegand et al. [33], who noted that PIK3CA and ARID1A are more frequently mutated in clear cell and endometrioid ovarian cancers, which are subtypes that generally lack CCNE1 amplification.

Integrative single-cell and spatial transcriptomic analyses revealed subtype-specific roles of CCNE1 in ovarian cancer. In both clear cell ovarian carcinoma (CCOC) and high-grade serous ovarian carcinoma (HGSOC), CCNE1 expression is largely confined to epithelial cells, supporting previous bulk genomic findings [30, 34] that identified CCNE1 as a lineage-specific driver of ovarian tumors proliferation. This finding aligns with broader oncological evidence linking CCNE1 to aggressive tumor behavior. Pan-cancer analyses [35] have shown that CCNE1 is frequently overexpressed across various malignancies and is associated with poor prognosis and proliferation-related pathways, including cell cycle progression and DNA replication. Importantly, the relationship between CCNE1 and immune infiltration appears to be highly context dependent. In our datasets, CCNE1^high^ regions lacked strong spatial correlation with immune markers (such as PDCD1 and CD8A), a pattern consistent with recent findings suggesting that CCNE1-driven tumors may engage in immune evasion not by actively excluding immune cells but by lacking proinflammatory cues.

The enrichment of focal adhesion pathways in CCNE1^high^ cells has important mechanistic implications. Focal adhesion kinase (FAK), a central node in this pathway, has been identified as a key mediator of tumor immune evasion. It orchestrates the recruitment of immunosuppressive regulatory T cells (Tregs) and dampens cytotoxic T-cell activity by modulating chemokine networks [36]. While we did not observe overt immune exclusion in CCNE1^high^ regions, it is plausible that FAK-mediated structural remodeling contributes to an immune-desert phenotype via altered stromal–immune cell communication. Additionally, the spatial colocalization of CCNE1 with ARHGAP1 and STK24, both of which are implicated in cytoskeletal regulation, echoes findings from studies of other tumors in which cell motility pathways enable immune evasion. For example, in breast cancer [37], alterations in focal adhesion signaling through Crk adaptor proteins promote PD-L1 expression and epithelial-mesenchymal transition (EMT), further shielding tumors from immune detection. Additionally, the spatial colocalization of CCNE1 with ARHGAP1 and STK24, both of which are involved in cytoskeletal and adhesion regulation, may reflect an immunomodulatory mechanism through structural reprogramming. ARHGAP family members shape a tumor-promoting microenvironment by facilitating T-cell exhaustion and increasing regulatory T-cell (Treg) infiltration, particularly in bladder and renal cancers [38]. Recent studies [39] have highlighted STK24 as a key immune evasion mediator. STK24 directly phosphorylates AKT, increases PD-L1 expression and enables resistance to cytotoxic T cell responses. Although our findings suggest that CCNE1 may promote an immune-desert phenotype through activation of structural remodeling pathways such as FAK, ARHGAP1, and STK24, these associations are based on transcriptomic correlations rather than direct functional assays. Moreover, replication stress resulting from CCNE1 amplification may suppress the cGAS–STING pathway, diminishing innate immune activation and contributing to the immune-desert phenotype observed in this study. Future experimental validation, such as genetic perturbation or pharmacologic inhibition of FAK/ARHGAP1 in CCNE1-high models with readouts of T-cell infiltration and cytotoxicity, will be essential to confirm their causal role in immune modulation. The observed immune-desert phenotype of CCNE1^high^ tumors underscores their limited responsiveness to immune checkpoint blockade. Future studies integrating CCNE1 status with immunotherapy biomarkers may help identify subgroups that require combination strategies targeting both cell-cycle and immune pathways.

Given the frequent associations of CCNE1 amplification with poor prognosis, platinum resistance, and homologous recombination proficiency (HRP) in gynecologic malignancies, emerging evidence supports a multipronged therapeutic strategy. One potential avenue involves the direct pharmacological inhibition of CCNE1 by targeting CDK2-dependent functions. For example, preclinical studies (NCT05252416) with BLU-222 demonstrated low nanomolar GI₅₀ values, G₁ cell cycle arrest, hypophosphorylation of retinoblastoma protein, and robust tumor regression in CCNE1^high^ ovarian and endometrial xenografts [17]. Similarly, INX-315, a next-generation CDK2 inhibitor, induced cell cycle arrest and senescence in CCNE1-amplified cancer models with durable tumor control [40]. In addition, adavosertib achieved a 36% objective response rate and a 6.3-month median PFS in CCNE1-amplified, platinum-resistant HGSOC by exploiting replication stress [13]. Adavosertib continues to be evaluated in multiple phase II trials, including NCT04590248 and NCT04266912, which explore its efficacy in gynecologic malignancies.

Another strategy leverages the HR-proficient phenotype of CCNE1-amplified tumors. In this context, combination therapies using WEE1 or ATR inhibitors along with platinum agents or PARP inhibitors have been explored to induce synthetic lethality and enhance treatment sensitivity [13]. Moreover, recent studies have suggested that reprogramming the tumor immune microenvironment may enhance the responsiveness to immune checkpoint inhibitors. Specifically, CDK4/6 inhibitors not only suppress tumor cell proliferation but also induce T cell-mediated inflammatory responses, enhance antigen presentation, and upregulate PD-L1 expression. These effects convert immunologically “cold” tumors into “hot” tumors, leading to robust synergistic antitumor activity when combined with PD-L1 inhibitors, as demonstrated in multiple murine models [42–44]. Similarly, STING agonists activate the cGAS-STING signaling pathway, promoting the polarization of tumor-associated macrophages toward an M1-like phenotype, activating dendritic cells, and increasing the infiltration of CD8^+^ T and natural killer (NK) cells [45]. These immunomodulatory effects have been shown to prolong survival in preclinical models and further enhance efficacy when administered in combination with an immune checkpoint blockade [46].

## Strengths and limitations

The strength of our study lies in its multimodal and integrative design, which combines bulk CNA analysis, single-cell transcriptomics, and spatial transcriptomics across cervical, endometrial, and ovarian cancers. However, several limitations must be acknowledged. First, our analysis relies on retrospective data aggregated from public repositories with diverse sequencing platforms and bioinformatics pipelines, which may introduce variability in CNA calling and expression quantification. Moreover, although our integrative analysis revealed associations between CCNE1 amplification and structural or signaling features, it lacked definitive functional validation. Additionally, clinical information such as direct clinical outcomes and treatment responses was unavailable in our dataset, preventing analysis of the impact of CCNE1 amplification on oncologic outcomes. Future experimental validation is required to confirm causal relationships. CRISPR-mediated CCNE1 perturbation and patient-derived endometrial xenograft models could clarify the effects of CCNE1 on tumor growth, immune infiltration, and sensitivity to CDK2 or WEE1 inhibitors.

## Conclusion

CCNE1 amplification is most prevalent in high-grade serous ovarian carcinoma and serous endometrial carcinoma but is rare in cervical and low-grade tumors. It frequently cooccurs with TP53 and PI3K-AKT pathway alterations and is largely confined to epithelial cells with minimal immune infiltration. Functional analyses suggested its role in promoting proliferation, cell adhesion, and structural remodeling. CCNE1 amplification reflects molecular features of aggressive gynecological cancer subtypes and may inform patient stratification in targeted therapy studies, though its predictive utility requires further clinical validation.

## Supplementary Information

Below is the link to the electronic supplementary material.

Supplementary Material 1.

## Data Availability

Most of the raw data are included in the Supplementary Materials. Data and reagents are available from the corresponding author upon reasonable request. The genomic data from the GENIE dataset used in this study are openly available for download via cBioPortal ( [https://www.cbioportal.org](https:/www.cbioportal.org) ). The RNA-seq data are publicly available from the GEO repository under accession codes GSM235931, GSE197461, and GSE173682.
