# Supplementary material for: Multiomic analysis of CCNE1 amplification associated molecular and immune features in gynecological cancers
Source: Discov Oncol. 2025 Dec 3;16:2225. doi: 10.1007/s12672-025-04032-7 (PMC12748378; doi:10.1007/s12672-025-04032-7)
Supplement: Supplementary file 1 — Supplementary Material 1. [file 12672_2025_4032_MOESM1_ESM.pdf]

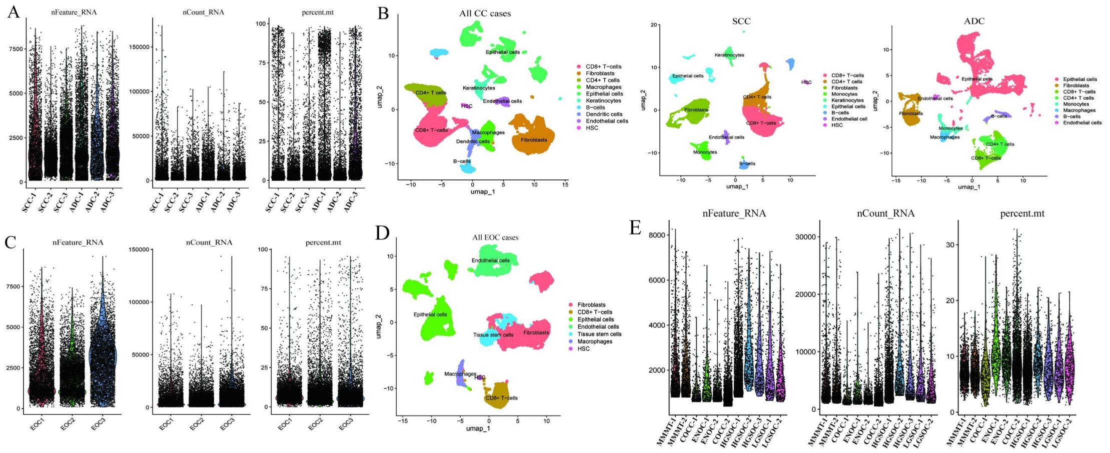

Supplementary Figure S1. Single-cell transcriptomic analysis across cervical, endometrial, and ovarian cancers. (A) Quality control metrics for cervical cancer samples (SCC and ADC), including number of detected genes, total RNA counts, and mitochondrial RNA percentage. (B) t-SNE plots showing major cell type clusters identified via SingleR in all cervical cancer cases, and separately in SCC and ADC samples. (C) Quality control metrics for endometrial cancer samples showing gene count and mitochondrial content. (D) UMAP plot displaying cell clusters annotated into seven major cell types in endometrial cancer. (E) Quality control metrics for ovarian cancer subtypes (HGSOC, LGSOC, CCOC, ENOC, and MMMT), including gene counts, total RNA, and mitochondrial RNA content.

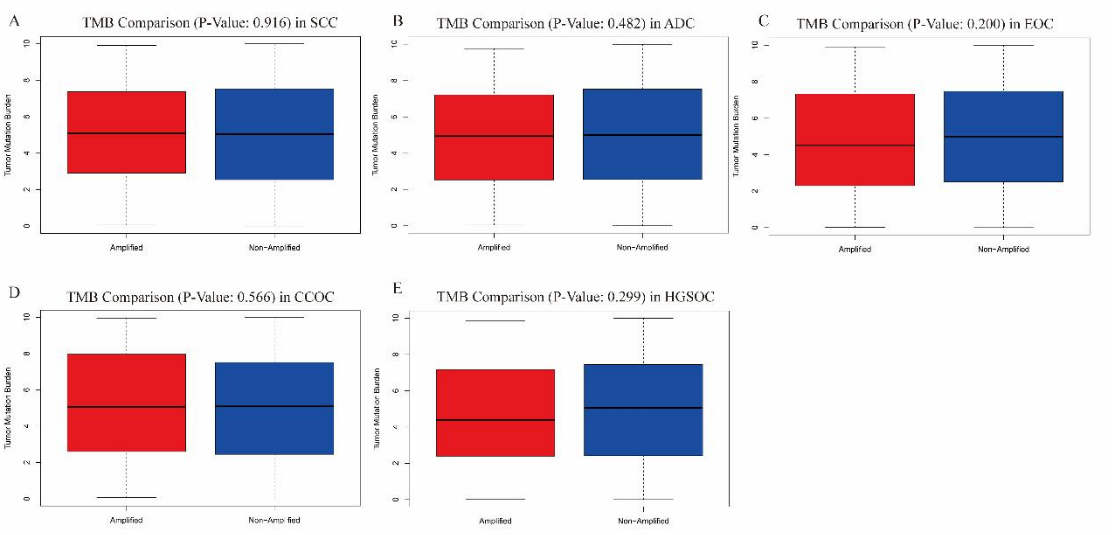

Supplementary Figure S2. Comparison of tumor mutational burden (TMB) between CCNE1-amplified and non-amplified samples across gynecologic cancer subtypes. (A-E) Boxplots comparing total mutation burden between CCNE1-amplified and non-amplified tumors in (A) squamous cell carcinoma (SCC), (B) adenocarcinoma (ADC), (C) endometrioid ovarian cancer (EOC), (D) clear cell ovarian cancer (CCOC), and (E) high-grade serous ovarian cancer (HGSOC). No statistically significant differences were observed across subtypes (all $P > 0.05$).

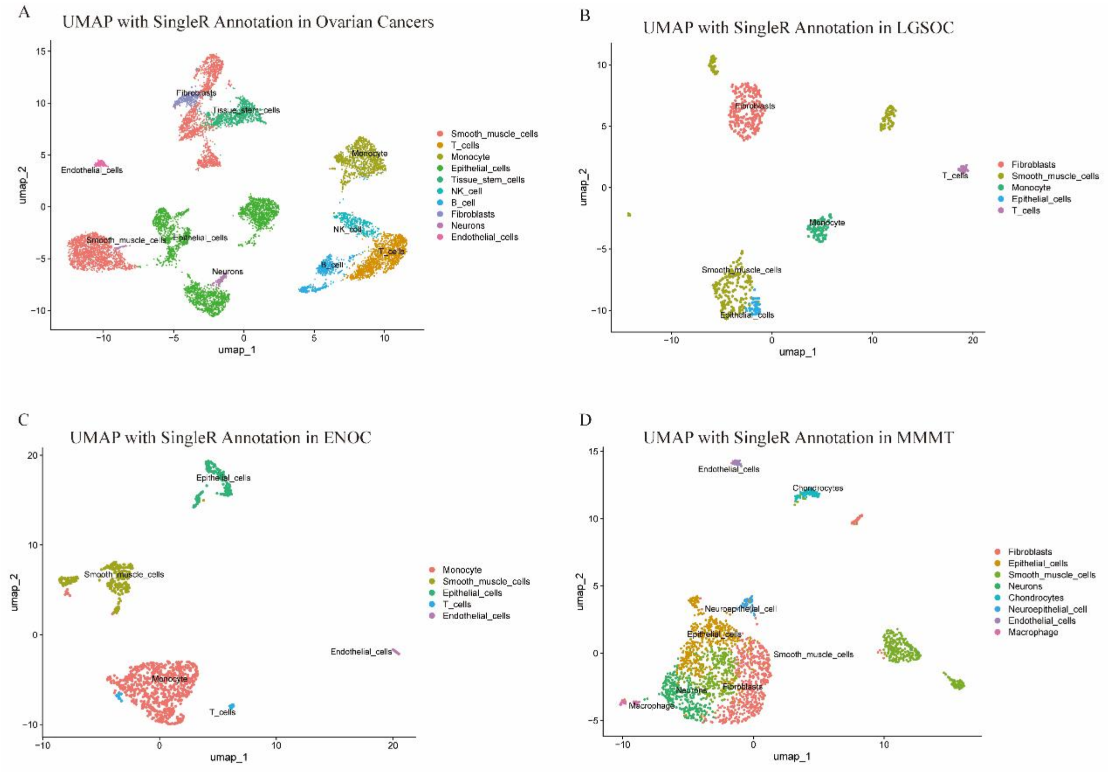

Supplementary Figure S3. Cell type annotation of single-cell transcriptomes across ovarian cancer subtypes. (A–D) UMAP visualization of cell clusters in ovarian cancer samples annotated using the SingleR package. Cell types were identified following dimensionality reduction and clustering of scRNA-seq data from (A) all ovarian cancer samples, and specifically from (B) low-grade serous ovarian cancer (LGSOC), (C) endometrioid ovarian cancer (ENOC), and (D) ovarian carcinosarcoma (MMMT).

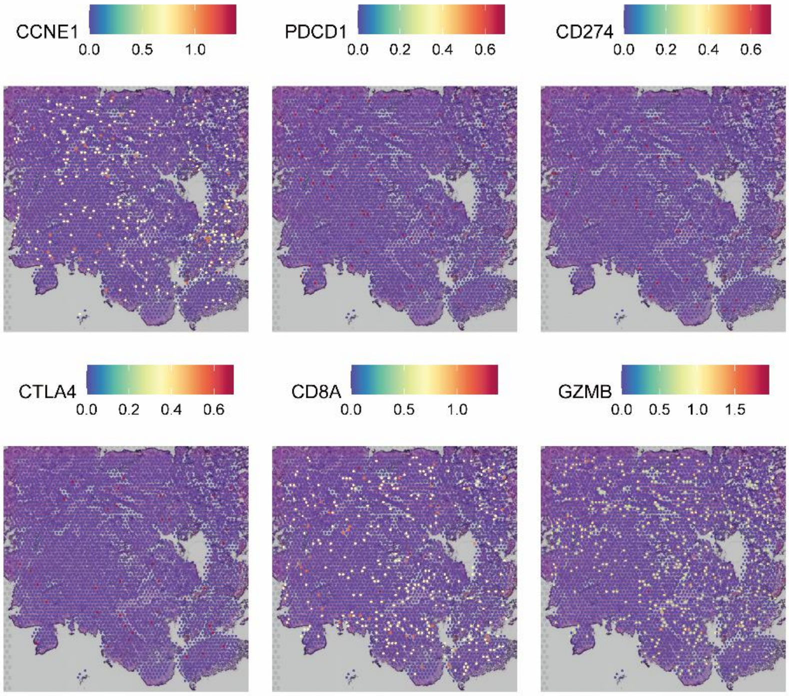

Supplementary Figure S4. Spatial transcriptomic visualization of CCNE1 and immune-related

gene expression. Spatial feature plots display the expression patterns of CCNE1 and key immune markers (PDCD1, CD274, CTLA4, CD8A, and GZMB) in high-grade serous ovarian carcinoma (HGSOC) tissue sections.
